# Supplementary material for: Ageing is associated with disrupted reinforcement learning whilst learning to help others is preserved
Source: Nat Commun. 2021 Jul 21;12:4440. doi: 10.1038/s41467-021-24576-w (PMC8295324; doi:10.1038/s41467-021-24576-w)
Supplement: Supplementary file 3 — Reporting summary [file 41467_2021_24576_MOESM3_ESM.pdf]

nature research

Corresponding author(s): Dr Jo Cutler, Dr Patricia Lockwood

Last updated by author(s): Jun 3, 2021

Reporting Summary

Nature Research wishes to improve the reproducibility of the work that we publish. This form provides structure for consistency and transparency in reporting. For further information on Nature Research policies, see our Editorial Policies and the Editorial Policy Checklist.

Statistics

For all statistical analyses, confirm that the following items are present in the figure legend, table legend, main text, or Methods section.

n/a	Confirmed	
☐	☒	The exact sample size (*n*) for each experimental group/condition, given as a discrete number and unit of measurement
☐	☒	A statement on whether measurements were taken from distinct samples or whether the same sample was measured repeatedly
☐	☒	The statistical test(s) used AND whether they are one- or two-sided<br>*Only common tests should be described solely by name; describe more complex techniques in the Methods section.*
☐	☒	A description of all covariates tested
☐	☒	A description of any assumptions or corrections, such as tests of normality and adjustment for multiple comparisons
☐	☒	A full description of the statistical parameters including central tendency (e.g. means) or other basic estimates (e.g. regression coefficient) AND variation (e.g. standard deviation) or associated estimates of uncertainty (e.g. confidence intervals)
☐	☒	For null hypothesis testing, the test statistic (e.g. *F*, *t*, *r*) with confidence intervals, effect sizes, degrees of freedom and *P* value noted<br>*Give P values as exact values whenever suitable.*
☐	☒	For Bayesian analysis, information on the choice of priors and Markov chain Monte Carlo settings
☐	☒	For hierarchical and complex designs, identification of the appropriate level for tests and full reporting of outcomes
☐	☒	Estimates of effect sizes (e.g. Cohen's *d*, Pearson's *r*), indicating how they were calculated

Our web collection on statistics for biologists contains articles on many of the points above.

Software and code

Policy information about availability of computer code

Data collection	Presentation version 17 (Neurobehavioral Systems – https://www.neurobs.com/)
Data analysis	Custom scripts in MATLAB 2019b (including using SPM 8 and VBA toolbox) and R version 3.6.2 (with R studio; including packages: robustlmm, lme4, psych, rstatix, mediation, MASS). All scripts are available at https://doi.org/10.17605/osf.io/xgw7h/. Bayes factors calculated using JASP version 0.14.1.

For manuscripts utilizing custom algorithms or software that are central to the research but not yet described in published literature, software must be made available to editors and reviewers. We strongly encourage code deposition in a community repository (e.g. GitHub). See the Nature Research guidelines for submitting code & software for further information.

Data

Policy information about availability of data

All manuscripts must include a data availability statement. This statement should provide the following information, where applicable:

- Accession codes, unique identifiers, or web links for publicly available datasets
- A list of figures that have associated raw data
- A description of any restrictions on data availability

The data generated in this study are available on the Open Science Framework at: https://doi.org/10.17605/osf.io/xgw7h/. Source data are provided with this paper.

Field-specific reporting

Please select the one below that is the best fit for your research. If you are not sure, read the appropriate sections before making your selection.

- [ ] Life sciences
- [x] Behavioural & social sciences
- [ ] Ecological, evolutionary & environmental sciences

For a reference copy of the document with all sections, see nature.com/documents/nr-reporting-summary-flat.pdf

Behavioural & social sciences study design

All studies must disclose on these points even when the disclosure is negative.

Study description	The study analyses quantitative data, mainly from an experimental mixed design with the between-subject factor age-group (young / older) and within-subject factor of recipient condition (self / other / no one) in the prosocial learning task. Additional measures are from questionnaires and neuropsychological tests.
Research sample	75 young (aged 18-36, mean=23.07, 44 females) and 77 older (aged 60-80, mean=69.84, 40 females) adults carefully matched on gender, years of education, and IQ were included in the main analysis (see below). The samples were not intended to be representative of any specific population. The sample age ranges were chosen to cover early adulthood, starting just after the end of adolescence, and older adulthood.
Sampling strategy	The sampling strategy was self-selection as participants signed up by responding to the adverts circulated via university databases, including volunteers who are students and members of the community, social media, and adverts in local newspapers. We required the sample to consist of two age groups - young and older - matched on years of education and IQ. Therefore inclusion in the sample was partially determined by whether a participant contributed toward this matching. Sample size was determined as the maximum number within logistical constraints but the sample recruited gave 88% power to detect a medium size effect (d=0.5).
Data collection	Data were collected on a computer for the prosocial learning task and Self-Report Psychopathy questionnaire. A member of the research team (AA, LH, DD or PL) was present for this data collection but did not observe the participant's responses while completing these measures. The Wechsler Test of Adult Reading was also administered by a member of the research team. The Wechsler Test of Adult Reading was recorded by the researcher on paper. Researchers could not be blind to age-group condition but did not observe the participants completing the prosocial learning task in the different conditions.
Timing	14th September 2016 - 27th August 2019
Data exclusions	80 participants in each age group were recruited. Five young and three older participants were excluded due to: diagnosis of a psychiatric disorder at the time of testing (1 young participant); previous study of psychology (2 young participants); and incomplete or low-quality data (2 young and 3 older participants). This left a final sample of 152 participants. Two further older participants had learning rates more than 3 standard deviations above the mean so were excluded from those analyses. An additional participant in each age group was missing data on the self-reported psychopathic traits measure.
Non-participation	No participants dropped out after starting the study.
Randomization	Groups were determined by age so naturally occurring. The groups were matched on gender, years of education, and IQ and we controlled for age-standardised intelligence in our control analyses. The recipient condition factor was manipulated within-subjects but the order of these conditions was randomised for each participant.

Reporting for specific materials, systems and methods

We require information from authors about some types of materials, experimental systems and methods used in many studies. Here, indicate whether each material, system or method listed is relevant to your study. If you are not sure if a list item applies to your research, read the appropriate section before selecting a response.

Materials & experimental systems

n/a	Involved in the study	
x		Antibodies
x		Eukaryotic cell lines
x		Palaeontology and archaeology
x		Animals and other organisms
	x	Human research participants
x		Clinical data
x		Dual use research of concern

Methods

n/a	Involved in the study	
x		ChIP-seq
x		Flow cytometry
x		MRI-based neuroimaging

Human research participants

Policy information about studies involving human research participants

Population characteristics	Please see above.
Recruitment	We recruited 80 young participants and 80 older participants using the same recruitment methods in order to match the samples as closely as possible. Participants were recruited from university databases, which included students and members of the community, social media, and adverts in local newspapers. While our age groups were matched on years of education and IQ, the recruitment from university databases or issues around self-selection may mean that the levels of education and IQ in our sample are not completely representative of the general population. This self-selection bias could impact results by meaning that the age-related differences we observe only apply to people with levels of education and IQ within the range of our participants. However, controlling for these measures did not change our results. We highlight this point in our discussion.
Ethics oversight	Oxford University Medical Sciences Inter Divisional Research Ethics Committee and National Health Service Ethics

Note that full information on the approval of the study protocol must also be provided in the manuscript.
